# A Severely Dilated Gallbladder With Multiple Gallstones After Concomitant Laparoscopic Sleeve Gastrectomy and Childbirth in a Hispanic Woman

**DOI:** 10.7759/cureus.42963

**Published:** 2023-08-04

**Authors:** Lord Mvoula, Tarek Khrisat, Sherry Melton

**Affiliations:** 1 Surgery, Lincoln Medical and Mental Health Center, Bronx, USA

**Keywords:** gallbladder, bariatric, laparoscopic, nonalcoholic fatty liver disease, s: cholelithiasis

## Abstract

Independent studies have associated laparoscopic sleeve gastrectomy (LSG) and pregnancy with a higher incidence of gallstones, especially in Hispanic populations. However, the synergistic impact of these risk factors is currently unknown. We present the case of a 42-year-old Hispanic woman who initially identified intermittent upper right abdominal pain, which worsened over the last four days before the presentation. Abdominal ultrasound indicated hepatic steatosis, hepatomegaly, and cholelithiasis. A hydropic gallbladder with numerous gallstones, surrounding pericholecystic inflammatory changes, and mild intra-abdominal and pelvic ascites was confirmed by computed tomography. The patient underwent an uneventful robotic-assisted cholecystectomy. A gross examination of the gall bladder measuring 15.5 x 6 x 5.5 cm revealed multiple stones measuring 1.0-1.5 cm in the lumen, the largest of which was impacted in the neck. This case underscores the importance of considering ethnicity and pregnancy history while assessing the post-LSG risk of incident cholelithiasis.

## Introduction

Rapid weight loss after bariatric surgery (BS) may severely impair gallbladder emptying and induce gallstone formation [1]. Despite obesity being a well-known ailment with a recognized impact on morbidity and mortality, the direction of treatment has largely stayed the same and is centered on weight loss. Today, Roux-en-Y gastric bypass (RYGB), sleeve gastrectomy (SG), and adjustable gastric banding are the most popular and commonly performed BSs [2].

Laparoscopic sleeve gastrectomy (LSG) is associated with incident gallstone formation with cholelithiasis in 23-48% of the patients [3,4]. However, only about a fourth of these patients have complicated cholelithiasis, of which most undergo cholecystectomy between nine months and 23 months post-LSG [3]. For instance, 6.8% of patients undergo cholecystectomy after RYGB [5]. Furthermore, gallstone formation is also common during pregnancy, with a 3-12% incidence rate [6].

Here, we report a case of complicated cholelithiasis four months after concomitant LSG and childbirth in a Hispanic patient, highlighting the necessity to consider ethnicity and pregnancy history while assessing the post-LSG risk of incident cholelithiasis.

## Case presentation

A 42-year-old Hispanic woman identified upper right abdominal pain over the past week, which was intermittent initially but worsened over the last four days before the presentation. The pain was accompanied by nausea, poor appetite, subjective fevers, chills, abdominal tenderness in the right upper quadrant without guarding or rebounding, and positive for Murphy's sign. The patient reported no chest pain, new cough, or dysuria. The frequency of urination and defecation was as usual.

The patient had a significant past medical history of anemia, asthma, hypothyroidism, mixed hyperlipidemia, multiseptated hypovascular right ovarian cyst measuring about 4.8 x 3.1 x 3.5 cm, menorrhagia, remote appendectomy, and LSG in 2020, followed by gastric bypass with RYGB in 2022, five days prior to her last pregnancy or four months after delivery. The patient did not have a history of any allergies or tobacco, alcohol, vaping, and illicit drug use.

During the current visit, the patient weighed 95 kg (BMI=31 kg/m^2^). Pre-surgery laboratory tests showed a high WBC count of 16.1 x 10^3^/mcL (reference range: 4.80-10.80 x 10^3^/mcL), alanine transaminase 172 U/L (reference range: ≤33), aspartate transaminase 206 U/L (reference range: ≤32), alkaline phosphatase 344 U/L (reference range: 35-105), total bilirubin 1.81 mg/dL (reference range: 0.2-1.2), and low hematocrit 36.6% (reference range: 37.0-47.0%). Other laboratory parameters (hemoglobin, platelet count, serum albumin, total protein, blood urea nitrogen, creatinine, and blood electrolytes) were within the clinical range (Table 1).

**Table 1:** Blood biochemistry of the patient pre- and post-surgery. POD: postoperative day. Values in bold indicate that they are outside the reference range.

Variable	Pre-surgery	POD1	Reference range
Complete blood count			
WBC count, x10^3^/mcL	16.10	18.17	4.80-10.80
Hemoglobin, g/dL	12.1	10.4	12.0-16.0
Hematocrit, %	36.6	33.3	37.0-47.0
Platelet count, x10^3^/mcL	299	288	150-450
Liver function test			
Alanine transaminase, U/L	172	148	≤33
Aspartate transaminase, U/L	206	113	≤32
Alkaline phosphatase, U/L	344	251	35-105
Total bilirubin, mg/dL	1.81	0.5	0.2–1.2
Albumin, g/dL	3.7	3.2	3.5-5.2
Total protein, g/dL	6.7	6.2	6.4-8.3
Kidney function test			
Blood urea nitrogen, mg/dL	11.0	18.0	6-24
Creatinine, mg/dL	0.70	1.08	0.59-1.04
Blood electrolytes			
Sodium, mmol/L	139	140	135-145
Potassium, mmol/L	4.3	4.6	3.5-5.3
Phosphorus, mg/dL	4.4	4.0	2.5-4.5
Magnesium, mg/dL	1.90	2.00	1.6-2.6
Glucose, mg/dL	99	110	70-99

**Figure 1:**
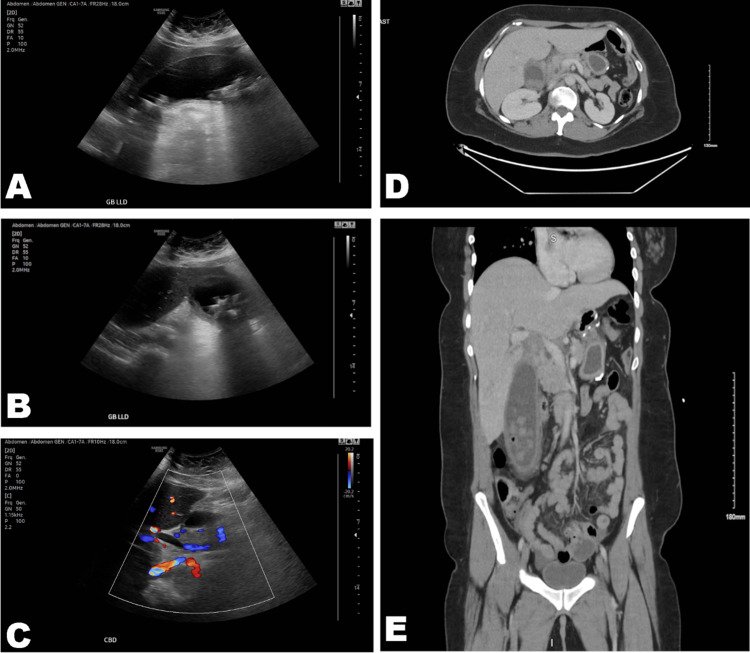
Ultrasound and computed tomography scans. (A, B) An oblique sonogram shows an enlarged gallbladder with several gallstones. (C) A color Doppler sonogram shows a common bile duct of 8 mm. (D, E) Computed tomography scans show an enlarged gallbladder with several gallstones and hepatomegaly.

No cardiovascular, respiratory, musculoskeletal, or neurological distress was observed during the physical examination. Abdominal ultrasound indicated hepatic steatosis, hepatomegaly, and severe cholelithiasis (Figure 1). A hydropic gallbladder with numerous gallstones, surrounding pericholecystic inflammatory changes, and mild intra-abdominal and pelvic ascites was confirmed by computed tomography (Figure 1).

**Figure 2:**
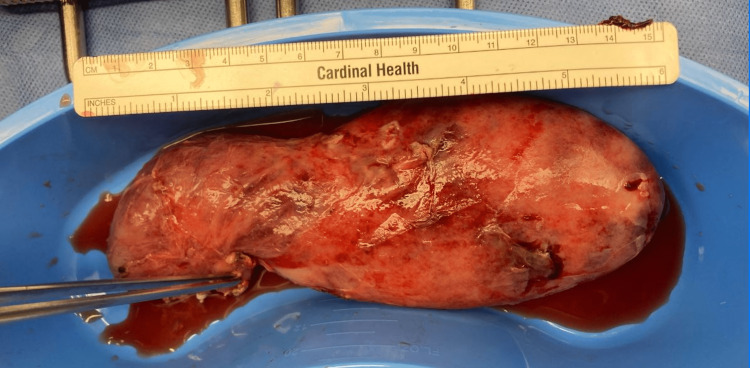
A highly distended gallbladder measuring 15.5 x 6 x 5.5 cm.

The patient underwent an uneventful robotic-assisted cholecystectomy with an intraoperative cholangiogram performed by a surgeon who has 32 years of experience with cholecystectomy. One reactive benign lymph node measuring 2.0 cm in diameter with focal acute inflammation of the surrounding tissue was also removed. A gross examination of the gall bladder measuring 15.5 x 6 x 5.5 cm revealed multiple stones measuring 1.0-1.5 cm in the lumen, the largest of which was impacted in the neck (Figure 2). The visceral surface was partially smooth and partially rough, with finely congested blood vessels over its surface. The wall thickness was up to 1.1 cm. All intraoperative and postoperative pain control medications were administered, considering the patient was breastfeeding. The patient was discharged for home recovery on the third postoperative day (POD).

## Discussion

The patient's medical history and diagnosis were consistent with previously published literature. The incidence of gallstones has been positively associated with BMI, >40 years of age, female sex, and hyperlipidemia [7,8]. Similarly, the hepatobiliary-related findings of ultrasonography and computed tomography in our patient were consistent with previous studies, which indicate a strong association between gallstones and nonalcoholic fatty liver disease [9]. Indeed, most studies comment on this relationship; however, very few studies report on the gallbladder size post RYGB. This patient had a prior scan with a normal gallbladder in 2021, one year after her LSG. Thus, this case report is the only one to note this relationship.

In addition to age, gender, comorbid metabolic disorders, and LSG, pregnancy is a well-known risk factor for cholelithogenesis. The incidence of gallstones during pregnancy may range from 1.2% to 6.3%, with higher incidences with every additional pregnancy [8]. The incidence of biliary sludge formation, a necessary precursor for cholelithogenesis, is even higher during pregnancy, ranging from 3.2% to 15% [8]. Moreover, several studies have identified gallbladder disease, including gallstones, as a major nonobstetric cause of maternal morbidity and hospitalization during pregnancy and within one year postpartum, with a disproportionately high risk among Hispanic populations [8,10,11].

This increased lithogenicity has been attributed to estrogen-induced cholesterol biosynthesis, supersaturation of bile with cholesterol, biliary hypersecretion, and partial emptying of the gallbladder during pregnancy, collectively contributing to an increased risk of cholelithogenesis [8]. Although there is a lack of direct evidence, it is likely that our patient's ethnicity and history of LSG and pregnancy synergistically contributed to the risk and pathogenesis of cholelithiasis. However, if the potentially synergistic effect also resulted in an abnormally large gallbladder seen in our patient, this warrants further investigation. Our case report is one of the few, if not the only one, that highlights the relationship between gallbladder size and a history of RYGB.

Studies have shown that concomitant laparoscopic cholecystectomy with SG is safe and minimizes the need for a second surgery for symptomatic cholelithiasis [5,12]. Similarly, while gallstones with or without cholecystitis may significantly increase the risk of preterm birth, maternal morbidity, and maternal or neonatal rehospitalization, antepartum cholecystectomy was associated with reduced maternal rehospitalization [13]. Furthermore, a meta-analysis by Athwal et al. [14] showed that antepartum cholecystectomy did not increase the risk of preterm labor and fetal or maternal mortality.

Our findings may also have immediate clinical relevance in screening patients with a higher risk of complicated cholelithiasis, which must be studied in well-powered controlled trials.

## Conclusions

Cholelithogenesis is affected by multiple factors, including age, gender, comorbid metabolic disorders, LSG, and pregnancy. Few studies comment on the gallbladder size in patients with symptomatic cholelithiasis who underwent LSG or RYBG. This case underscores that relationship and highlights the importance of considering ethnicity and pregnancy history while assessing the post-LSG risk of incident cholelithiasis.
